# Human T-lymphotropic virus type 1 (HTLV-1) and cellular immune response in HTLV-1-associated myelopathy/tropical spastic paraparesis

**DOI:** 10.1007/s13365-020-00881-w

**Published:** 2020-07-23

**Authors:** Satoshi Nozuma, Ryuji Kubota, Steven Jacobson

**Affiliations:** 1 Viral Immunology Section, Division of Neuroimmunology and Neurovirology, National Institute of Neurological Disorders and Stroke, National Institutes of Health, Bethesda, MD USA; 2 Division of Neuroimmunology, Joint Research Center for Human Retrovirus Infection, Kagoshima University, Kagoshima, Japan

**Keywords:** HTLV-1, HAM/TSP, Neurological disorders, Immunology, Pathogenesis

## Abstract

Human T-lymphotropic virus type 1 (HTLV-1) is associated with adult T cell leukemia/lymphoma and HTLV-1-associated myelopathy/tropical spastic paraparesis (HAM/TSP). HAM/TSP is an inflammatory disease of the spinal cord and clinically characterized by progressive spastic paraparesis, urinary incontinence, and mild sensory disturbance. The interaction between the host immune response and HTLV-1-infected cells regulates the development of HAM/TSP. HTLV-1 preferentially infects CD4^+^ T cells and is maintained by proliferation of the infected T cells. HTLV-1-infected cells rarely express viral antigens in vivo; however, they easily express the antigens after short-term culture. Therefore, such virus-expressing cells may lead to activation and expansion of antigen-specific T cell responses. Infected T cells with HTLV-1 and HTLV-1-specific CD8^+^ cytotoxic T lymphocytes invade the central nervous system and produce various proinflammatory cytokines and chemokines, leading to neuronal damage and degeneration. Therefore, cellular immune responses to HTLV-1 have been considered to play important roles in disease development of HAM/TSP. Recent studies have clarified the viral strategy for persistence in the host through genetic and epigenetic changes by HTLV-1 and host immune responses including T cell function and differentiation. Newly developed animal models could provide the opportunity to uncover the precise pathogenesis and development of clinically effective treatment. Several molecular target drugs are undergoing clinical trials with promising efficacy. In this review, we summarize recent advances in the immunopathogenesis of HAM/TSP and discuss the perspectives of the research on this disease.

## Introduction

Human T-lymphotropic virus type 1 (HTLV-1) is the human retrovirus firstly discovered in 1980 (Poiesz et al. 1980). The main highly endemic areas are the Southwestern part of Japan, the Caribbean, South America, Central and Southern Africa, Middle East, and Central Australia. It is estimated that at least five to ten million people are infected with HTLV-1, but the current number of HTLV-1 carries might be much higher due to a lack of systematic epidemiological studies in most endemic regions (Gessain and Cassar 2012). For example, it recently reported that more than 40% of central Australian Indigenous adults in some remote communities are HTLV-1c infected (Einsiedel et al. 2016). While the majority of infected individuals remain lifelong asymptomatic carriers (ACs), approximately 2–5% develop adult T cell leukemia/lymphoma (ATLL) (Uchiyama et al. 1977) and another 0.25–3.8% develop HTLV-1-associated myelopathy/tropical spastic paraparesis (HAM/TSP) (Gessain et al. 1985; Osame et al. 1986). HTLV-1 has also been associated with other inflammatory diseases including uveitis, myositis, infective dermatitis, and interstitial pneumonitis (Gessain and Mahieux 2012). HAM/TSP is clinically characterized by chronic progressive spastic paraparesis, urinary incontinence, and mild sensory disturbance. HTLV-1 preferentially infects CD4^+^CD25^+^ T cells in vivo and induces functional changes in the infected cells. HTLV-1-infected cells rarely express viral antigens in vivo; however, they easily express antigens after short-term culture. Therefore, such virus-expressing cells may lead to activation and expansion of antigen-specific T cell responses. The virus-host immunologic interaction plays a pivotal role in the pathogenesis of HAM/TSP. HTLV-1-infected T cells and CD8^+^ cytotoxic T lymphocytes (CTL) against HTLV-1 invade the central nervous system (CNS) and release proinflammatory cytokines and chemokines, resulting in the tissue damage. These immune responses are considered to underlie the pathogenesis of HAMTSP. Both HTLV-1 viral regulatory proteins, Tax and HTLV-1 basic leucine zipper factor (HBZ), play critical roles in immune dysregulation in HAM/TSP (Enose-Akahata et al. 2017). Tax induces the expression of many host cellular genes and consequently contributes to cell activation and proliferation. HBZ induces inflammation in the host through altering the Foxp3 expression in Treg cells (Satou et al. 2011; Yamamoto-Taguchi et al. 2013). Although HAM/TSP is not directly life-threatening, the disease severely impacts patients’ quality of life (Olindo et al. 2006; Coler-Reilly et al. 2016), and treatment remains highly unsatisfactory. Humanized mice models were developed and could be potential novel tools for understanding HTLV-1 neuropathogenesis and testing of novel therapies for HAM/TSP. Several trials for new drugs have been performed and show promising results. Here, we summarize the immunopathology of HAM/TSP, specifically focused on cell-mediated immunity in this disease and discuss ongoing developments and perspectives of HAM/TSP research.

## HTLV-1 virus

HTLV-1 belongs to the *Deltaretrovirus* genus of the *Orthoretrovirinae* subfamily of retroviruses. Most HTLV-1-infected T cells contain a single integrated provirus (Cook et al. 2012). The HTLV-1 proviral genome is 9 kb in length and contains structural genes, *gag*, *pol*, and *env* flanked by a long terminal repeat at both the 5′ and 3′ ends. The HTLV-1 genome also has a *pX* region including six open reading frames: five on the plus-strand and one on the minus-strand. Of these six genes, *tax*, *rex*, and *HBZ* play a key role in the infectivity and proliferation of HTLV-1. Other regulatory genes *p8*, *p12*, *p13*, and *p30* are expressed at very low levels and these proteins are considered to have minor roles in replication and persistence of HTLV-1 in vivo (Bangham 2018). HTLV-1 can be transmitted through intravenous drug use, sexual contact, and breastfeeding from mother to child. Familial clusters of HAM/TSP were reported (Nozuma et al. 2014; Alvarez et al. 2016), but genetic analysis of host genomes was not able to detect any disease-associated genes due to a small number of cases (Nozuma et al. 2017). HTLV-1 has remarkably low genetic variability both within and between hosts, although minor variations exist between geographical isolates (Komurian et al. 1991). Most studies of HTLV-1 genotype have reported no correlation between nucleotide substitutions and the risk of HAM/TSP (Mahieux et al. 1995), and the recent analysis of complete HTLV-1 sequence could not detect any HAM/TSP-specific mutations (Pessoa et al. 2014; Nozuma et al. 2017). However, the transcontinental HTLV-1 subtype is associated with an increased risk of HAM/TSP compared with the Japanese subtype in the Japanese population (Furukawa et al. 2000; Saito 2019). HAM/TSP patients with transcontinental subtype showed lower levels of *HBZ* mRNA expression (Yasuma et al. 2016a) and higher levels of CXCL10, which has been proposed to be a prognostic biomarker for HAM/TSP (Naito et al. 2018). A recent study showed that CTCF, a master regulator of chromatin structure and expression, bound to HTLV-1 and formed loops between proviral and host genomes to regulate the expression of proviral and host genes (Satou et al. 2016). Epigenetic modifications may regulate the pattern of proviral transcription and mediate inflammation in HAM/TSP patients, and further studies are necessary to clarify the role of these mechanisms in the pathogenesis of HAM/TSP.

### Molecular pathogenesis of Tax

The *tax* gene encodes a 353-amino acid, 40-kDa protein, Tax, that has a central role in HTLV-1 biology (Bangham 2018). HTLV-1 Tax is a transcriptional transactivator of virus replication and induces the expression of a variety of cellular genes by activation of the NF-κB and CREB/ATF pathways (Matsuoka and Jeang 2011). De novo infection requires expression of Tax since transcription of the sense-strand of the provirus which is responsible for the generation of the viral genome and viral proteins. Tax has been extensively studied because it induces the expression of many host cellular genes and consequently contributes to dysfunction in immune cells of HAM/TSP patients. Ex vivo, Tax protein is spontaneously expressed in peripheral blood mononuclear cells (PBMCs) after culture without any exogenous stimulation (Hanon et al. 2000a) and the level of *tax* mRNA was significantly higher in HAM/TSP patients than in ACs (Yamano et al. 2002). Tax boosts expression of T box transcription factor (T-bet), which promotes IFN-γ production (Araya et al. 2014). A number of the common γ chain family of cytokines and their receptors, such as IL-2/IL-2R, IL-9, IL-15/IL-15R, and IL-21/IL-21R have been demonstrated to be transactivated by Tax (Enose-Akahata et al. 2017).

Tax is an immunodominant antigen recognized by HTLV-1-specific cytotoxic CD8^+^ T cells (Jacobson et al. 1990). The number of Tax-specific CTL is greatly elevated and these CTL produce proinflammatory cytokines (Kubota et al. 1998) and show degranulation activity in HAM/TSP patients that is comparable with that in ACs (Abdelbary et al. 2011). Though Tax protein is usually undetected in vivo, recent analysis shows that it is expressed in intermittent but intense bursts at the single-cell level by the observation of HTLV-1-infected cell lines (Billman et al. 2017; Mahgoub et al. 2018). Therefore, Tax-specific immune responses are chronically activated and might be pathogenic, rather than protective, due to high cytotoxicity and the production of inflammatory cytokines leading to neural damage.

### Molecular pathogenesis and localization of HBZ

HBZ was first discovered in 2002 as a novel viral protein that contains an N-terminal transcription activation domain and a leucine zipper motif in its C-terminus (Gaudray et al. 2002). As HBZ closely cooperates with Tax, HBZ has opposing functions to Tax and modifies transcription of various host genes (Matsuoka and Jeang 2011). HBZ is persistently expressed in infected cells, maintains viral latency (Philip et al. 2014), and promotes proliferation of ATLL cells (Satou et al. 2006; Arnold et al. 2008), whereas Tax expression is frequently silenced. HBZ interacts with CREB/ATF pathway, suppresses Tax-mediated transactivation, and selectively inhibits the classical NF-κB pathway (Matsuoka and Jeang 2011). HBZ interacts with the tumor suppressor Rb, and promotes proliferation of infected cells and counteracts apoptosis through HBZ-induced expression of survivin (Kawatsuki et al. 2016). In HBZ-Tg mice, HBZ increases the number of CD4^+^Foxp3^+^ Treg cells and then converts them to Foxp3^−^ T cells with producing IFN-γ, which results in inflammation and tumors (Satou et al. 2011; Yamamoto-Taguchi et al. 2013). HBZ has been recently recognized to play a critical role in inflammation and pathogenesis of HAM/TSP. The level of *HBZ* mRNA detected in HAM/TSP patients is significantly lower than in ATLL patients but higher than in ACs. Furthermore, *HBZ* mRNA expression was associated with HTLV-1 proviral load and increased disease severity in HAM/TSP patients (Saito et al. 2009). Antibody response against HBZ was observed in HTLV-1-infected subjects and related to decreased CD4^+^ T cell activation in HAM/TSP patients (Enose-Akahata et al. 2013). HBZ is also an immunogenic protein recognized by HBZ-specific CTL clones; however, HBZ is regarded as a weaker immunogen for CTL than Tax. HBZ-specific CTL clones could not lyse ATLL cells (Suemori et al. 2009) and killed significantly fewer infected cells than were killed by Tax-specific CTL clones (Rowan et al. 2014). The weaker immunogenicity of HBZ could, therefore, allow HTLV-1-infected cells to escape from the host immune response.

HBZ contains nuclear localization signals in its central/bZIP domain and nuclear export signals in its N-terminus (Hivin et al. 2005). HBZ is found in the nucleus in leukemic cells and the function of nuclear HBZ has been thoroughly investigated and reported to interact with important transcription factors including CBP/p300, Smad3, p65, c-Jun, and forkhead family proteins especially in ATLL (Tanaka and Matsuoka 2018). However, HBZ protein has been recently reported to be localized in the cytoplasm of T cells depending on the expression of THEMIS (Kinosada et al. 2017). HBZ interferes with the complex formation of THEMIS with Grb2 and SHP-2, which results in inhibition of the suppressive functions of coinhibitory receptors TIGIT and PD-1, and subsequently might enhance activation of T cells. In HAM/TSP patients, HBZ is reported to localize exclusively in the cytoplasm of infected cells and the number of HBZ-positive cells is higher in HAM/TSP patients compared with ACs (Baratella et al. 2017; Forlani et al. 2019). Cytoplasmic HBZ was almost exclusively found in the CD4^+^ T cell without coexpression of CD25 and the coexpression of HBZ and Tax was rarely found in the same cell. Distinct subcellular compartments of HBZ might therefore be associated with different pathogenetic mechanisms observed in ATLL and HAM/TSP.

### HTLV-1-infected T cells

HTLV-1 preferentially infects CD4^+^ T cells in vivo and induces functional changes in the infected cells (Enose-Akahata et al. 2017; Yamano and Coler-Reilly 2017). An elevated HTLV-1 proviral load (PVL) is the main risk factor for developing HAM/TSP in HTLV-1-infected subjects and is strongly associated with disease pathogenesis (Nagai et al. 1998; Nagai et al. 2001). The receptors for HTLV-1 have been reported to involve HTLV-1 entry and include glucose transporter 1, neuropilin-1, and heparan sulfate proteoglycans (Jones et al. 2011). Since the HTLV-1 receptors are detected in almost all vertebrate cells, this virus can infect different types of cells in vitro (Hoshino 2012). However, HTLV-1-infected cells are associated with expression of several characteristic surface molecules including CD4, CD25, CCR4, and CADM1. HTLV-1 is predominantly transmitted by cell-to-cell contact via the virological synapse and creates new HTLV-1-infected T cell clones in each host (Bangham 2018). It is thought that HTLV-1 infects mature lymphocytes, macrophages, and dendritic cells in the periphery and is associated with chronic infection of this virus. Recent studies demonstrate that HTLV-1 infects hematopoietic stem cells (HSCs), and infected stem cells differentiate into diverse cell lineages (Furuta et al. 2017). However, only memory CD4^+^ T cells become major viral reservoirs and express distinct surface markers, where viral factors, such as HBZ and Tax, are thought to control infected cells during HSCs differentiation.

#### Regulatory T cells and phenotype

HTLV-1 infection of CD4^+^ T cells is associated with expression of several proteins that are characteristic of Treg cells, in particular CD25, FoxP3, and CCR4. Treg cells contribute to the maintenance of immunologic self-tolerance and play an important role in chronic viral infections. In HAM/TSP patients, CD4^+^CD25^+^ T cells contain higher amounts of HTLV-1 PVL and show higher levels of HTLV-1 *tax* mRNA expression than in CD4^+^CD25^−^ cells and produce various cytokines including INF-γ (Yamano et al. 2004). HTLV-1-infected CD4^+^CD25^+^ T cells are not functionally suppressive but rather are shown to stimulate and expand HTLV-1 Tax-specific CD8^+^ T cells (Yamano et al. 2004). Foxp3, a key transcription factor for Treg cells, is expressed in approximately 60% of ATLL cases and its function is regulated by HBZ. HBZ enhances TGF-β signaling and promotes FoxP3 expression, and impairs the suppressive function of Treg cells in HBZ-Tg mice (Satou et al. 2011; Zhao et al. 2011). In addition, HBZ increases the number of Treg cells and converts them to Foxp3^−^ T cells producing IFN-γ (Yamamoto-Taguchi et al. 2013). In HAM/TSP patients, the levels of the FoxP3 expression is decreased in CD4^+^CD25^+^ T cells compared with ACs and healthy controls (Yamano et al. 2005; Oh et al. 2006). Furthermore, in vitro transduction of Tax reduced the *FoxP3* mRNA expression and inhibited the suppressive function of the CD4^+^CD25^+^ T cells isolated from healthy donors (Yamano et al. 2005).

HTLV-1 preferentially infects cells expressing CCR4 and CD4 molecules (Hieshima et al. 2008). It is well recognized that CCR4 is specifically expressed in ATLL cells, but CCR4^+^ cells appear to suppressive the function of Treg cells (Bangham and Toulza 2011). HBZ-induced GATA3 expression in CD4^+^ T cells and subsequently enhanced transcriptional activity from the CCR4 promoter (Sugata et al. 2016). Upregulated CCR4 expression is associated with enhanced T cell migration and proliferation. In HAM/TSP patients, the frequency of IFN-γ-producing CD4^+^CD25^+^CCR4^+^ T cells is increased and correlated with disease activity and severity (Yamano et al. 2009). CD4^+^CCR4^+^ T cells which express the Th1 marker CXCR3 and produce T-bet and IFN-γ are present in the CNS (Araya et al. 2014). When stimulated by IFN-γ, astrocytes produce CXCL10 (one of the CXCR3 ligands), which recruits more CXCR3^+^ T cells, including infected cells, to the CNS (Ando et al. 2013). These cells also produce proinflammatory cytokines such as IFN-γ, which stimulates astrocytes, further creating an inflammatory positive feedback loop with subsequent tissue damage in the CNS (Yamano and Coler-Reilly 2017). Anti-CCR4 monoclonal antibody effectively reduced the proviral load and proinflammatory cytokines in PBMCs from patients with HAM/TSP (Araya et al. 2014; Yamauchi et al. 2015). Clinical trials of anti-CCR4 monoclonal antibody have been performed and showed promising results (Sato et al. 2018).

### Cytotoxic t lymphocytes

The cytotoxic CD8^+^ T cells are important for the elimination of virus-infected cells. The quality of the host’s CTL response to HTLV-1-infected cells play the dominant role in determining the set-point proviral load and the steady-state abundance of HTLV-1 replication (Bangham 2009). One of the most prominent features of the cellular immune response in HAM/TSP patients is that the number of HTLV-1-specific CTL is greatly elevated in PBMCs compared with ACs (Jacobson et al. 1990; Kubota et al. 2003). It is unknown why HAM/TSP patients show high proviral load in spite of their large number of CTL and whether or not the cytotoxic activity of HTLV-1-specific CTL is different between HAM/TSP patients and ACs. Recently, a functional CD8^+^ cell assay reveals no significant differences in CD8^+^ cell anti-viral efficacy between HAM/TSP patients and ACs (Asquith et al. 2005) and another functional study shows no differences in cytokine production and degranulation activity of Tax-specific CTL between the two groups (Abdelbary et al. 2011). More detailed studies are needed to investigate whether the total efficiency, but not the frequency, of HTLV-1 Tax-specific CTLs in vivo differs between HAM/TSP patients and ACs and how they contributes to the pathogenesis of HAM/TSP.

#### Tax-specific CTL

It has been shown that the most immunodominant HTLV-1 antigen recognized by HTLV-1-specific CTL is the Tax protein. The epitopes of Tax_11–19_ and Tax_301–309_ bind strongly to HLA-A*0201 and HLA-A*2402 respectively (Jacobson et al. 1990; Harashima et al. 2004). These virus-specific CTL produce proinflammatory cytokines and show degranulation activity (Kubota et al. 1998; Yamano et al. 2002; Abdelbary et al. 2011). Importantly, the CTL frequency is also much higher in CSF than in PBMCs and is proportional to the HTLV-1 PVL (Greten et al. 1998; Nagai et al. 2001). Tax-specific CTL are also detected in spinal cord parenchyma (Matsuura et al. 2015). Strong Tax-specific CTL response has been considered to control the viral replication and play a key role in the development of HAM/TSP. While Tax expression is generally decreased in infected cells in vivo (Hanon et al. 2000b), CTL response to Tax is chronically activated, suggesting frequent exposure to newly-synthesized Tax protein in vivo (Rende et al. 2011). A single cell analysis–clarified Tax protein was expressed in intermittent but intense bursts in HTLV-1-infected cells (Billman et al. 2017; Mahgoub et al. 2018). Recently, exosomes containing Tax was identified in virus-free CSF of patients with HAM/TSP and that in vitro, cells stimulated with Tax-containing exosomes became targets of HTLV-1 Tax-specific CTL (Anderson et al. 2018). These mechanisms may allow activated CTL to respond to persistently HTLV-1-infected cells, which may be associated with inflammatory tissue destruction.

#### HBZ-specific CTL

HTLV-1 Tax-specific CTL responses have been studied extensively; however, little is known about the frequency or function of HBZ-specific CTL in HAM/TPS patients. HBZ is also an immunogenic protein recognized by HBZ-specific CTL clones and HBZ-specific CTL have been identified in ATLL or HAM/TSP patients and ACs (Suemori et al. 2009; Macnamara et al. 2010). HTLV-1-infected individuals with HLA class I alleles strongly binding the HBZ protein were shown to be related to a lower proviral load and a reduced risk of HAM/TSP (Macnamara et al. 2010; Hilburn et al. 2011). However, compared with Tax-specific CTL, HBZ-specific CTL are at a lower frequency in the peripheral blood and kills fewer HTLV-1-infected cells in vitro (Macnamara et al. 2010; Rowan et al. 2014). HBZ is continuously expressed in HTLV-1-infected cells in vivo and HBZ induces the proliferation of these infected cells, thus indicating that HBZ might be a candidate antigen for cellular immunotherapy for HAM/TSP. It is necessary to identify HBZ epitopes or enhance anti-HBZ immune responses that can induce a stronger CTL response in HAM/TSP patients.

### Exhaustion and inhibitory receptor in HAM/TSP

Chronic viral infection has been reported to induce expression of inhibitory molecules that generate negative signals to downregulate the ensuing T cell responses. Recent translation of knowledge about inhibitory receptors such as CTLA-4 and PD-1 into cancer treatments highlights the opportunities to manipulate these pathways to treat human disease (Callahan et al. 2016). PD-1 is expressed on ATLL cells and CD8^+^ T cells in ATLL patients (Kozako et al. 2009; Yasuma et al. 2016b). Genomic analysis of ATLL demonstrated PD-L1 amplifications (Kataoka et al. 2018) and phase2 trial of PD-1 inhibitor therapy has been performed in patients with aggressive ATLL (Ishitsuka et al. 2018). In HAM/TSP patients, alternative expressions of various inhibitory receptors, such as PD-1, CD244, and Tim-3, have been demonstrated on CD8^+^ T cells (Enose-Akahata et al. 2009; Abdelbary et al. 2011; Kozako et al. 2011; Manuel et al. 2013). Some reports showed PD-1 expression is upregulated in both infected T cells and CTL (Yasuma et al. 2016b; Enose-Akahata et al. 2019) and these PD-1^+^ cells in CD8^+^ T cells showed CTL dysfunction in HAM/TSP patients (Manuel et al. 2013). T cell immunoglobulin and ITIM domain (TIGIT), which is another inhibitory molecule and expressed on activated T cells and Treg cells, are also highly expressed with PD-1 in HAM/TSP patients (Yasuma et al. 2016b). Combined blockade of PD-1 and TIGIT enhanced anti-Tax T cell response in PBMCs of HAM/TSP patients. Expression of multiple distinct inhibitory receptors is associated with greater T ell exhaustion and rapid disease progression and co-targeting multiple inhibitory receptors show synergy and substantially more robust reversal of T cell exhaustion and control of viral load in chronic viral infection (Attanasio and Wherry 2016). A combination of checkpoint-blocking antibodies may be a potential therapeutic treatment for HAM/TSP patients and may reduce HTLV-1-infected cells by recovering the function of CTL.

### T cell receptor repertoire analysis

T cell–mediated antigen recognition depends on the interaction of the T cell receptor (TCR) with the antigen-major histocompatibility complex (MHC) molecule. The diversity of TCR repertoires is central components of adaptive immune system function and can be altered in the context of infections, malignancies or immunological disorders. Analyzing TCR repertoires may help to gain a better understanding of immune-mediated responses in neuroinflammatory diseases. In HAM/TSP patients, a previous report on the TCR analysis showed a direct demonstration of clonal expansions within both CD4^+^ and CD8^+^ cells in HTLV-1 infections (Eiraku et al. 1998) and shared amino acid motif in the CDR3β in Tax-specific CTL in HLA-A*0201 HAM/TSP patients (Bourcier et al. 2001; Saito et al. 2001). With technological development based on high-throughput sequencing and bioinformatics methods, large-scale profiling of TCR repertoires have been conducted to characterize the structure of antigen-related TCRs, cancer-infiltrating T cells, and TCRs related to autoimmunity (Rosati et al. 2017). A recent analysis showed HAM/TSP patients had a higher clonal T cell expansion in PBMCs as well as purified CD4^+^ and CD8^+^ cells compared with multiple sclerosis (Alves Sousa et al. 2019). In addition, longitudinal analysis of TCR repertoires demonstrated a correlation of the TCR clonal expansion with HTLV-1 proviral load. ATLL patients also showed monoclonal TCRs compared with ACs, and clonality data observed based on TCR repertoires were completely consistent with clonality analysis based on provirus integration sites (Rowan et al. 2016; Farmanbar et al. 2019). Recently, Tax_301–309_-specific CTL in HLA-A*2402 ATLL patients and ACs shared highly restricted TCR repertoires by the single-cell analysis (Ishikawa et al. 2017). It is useful to identify expanded TCR clones of antigen-specific T cells and/or T cells found in the inflammatory lesions and clarify the function of these pathogenic T cells. We should target more antigen-specific T cells or compare TCR signatures between periphery and CNS to explore pathology-related TCR signature in HAM/TSP patients.

### Immunopathogenesis of HAM/TSP

The characteristic pathology of HAM/TSP consists of a chronic inflammation with diffuse degeneration throughout the central nervous system (Izumo et al. 2000). The spinal cord exhibits a loss of myelin and axons symmetrically in the lateral and posterior column with the inflammation of gray and white matter, which is dominant at the thoracic level (Iwasaki 1990; Yoshioka et al. 1993). These lesions are involved with perivascular and parenchymal lymphocytic infiltration with reactive astrocytosis and fibrillary gliosis (Umehara et al. 1993). Both CD4^+^ and CD8^+^ cells are evenly distributed in active inflammatory lesions, while the predominance of CD8^+^ cells and high levels of IFN-γ are detected in the chronic stage (Umehara et al. 1993; Aye et al. 2000). HTLV-1 mRNA and DNA are detected only in infiltrating CD4^+^ T cells in the spinal cord, but not in neural cells using in situ PCR technique (Moritoyo et al. 1996; Matsuoka et al. 1998). HTLV-1 has not been shown to actively infect neurons, oligodendrocytes, or microglia in vivo. These data strongly indicate that some of the infiltrating CD4^+^ T cells are infected with HTLV- 1, but the neural cells are not.

**Fig. 1:**
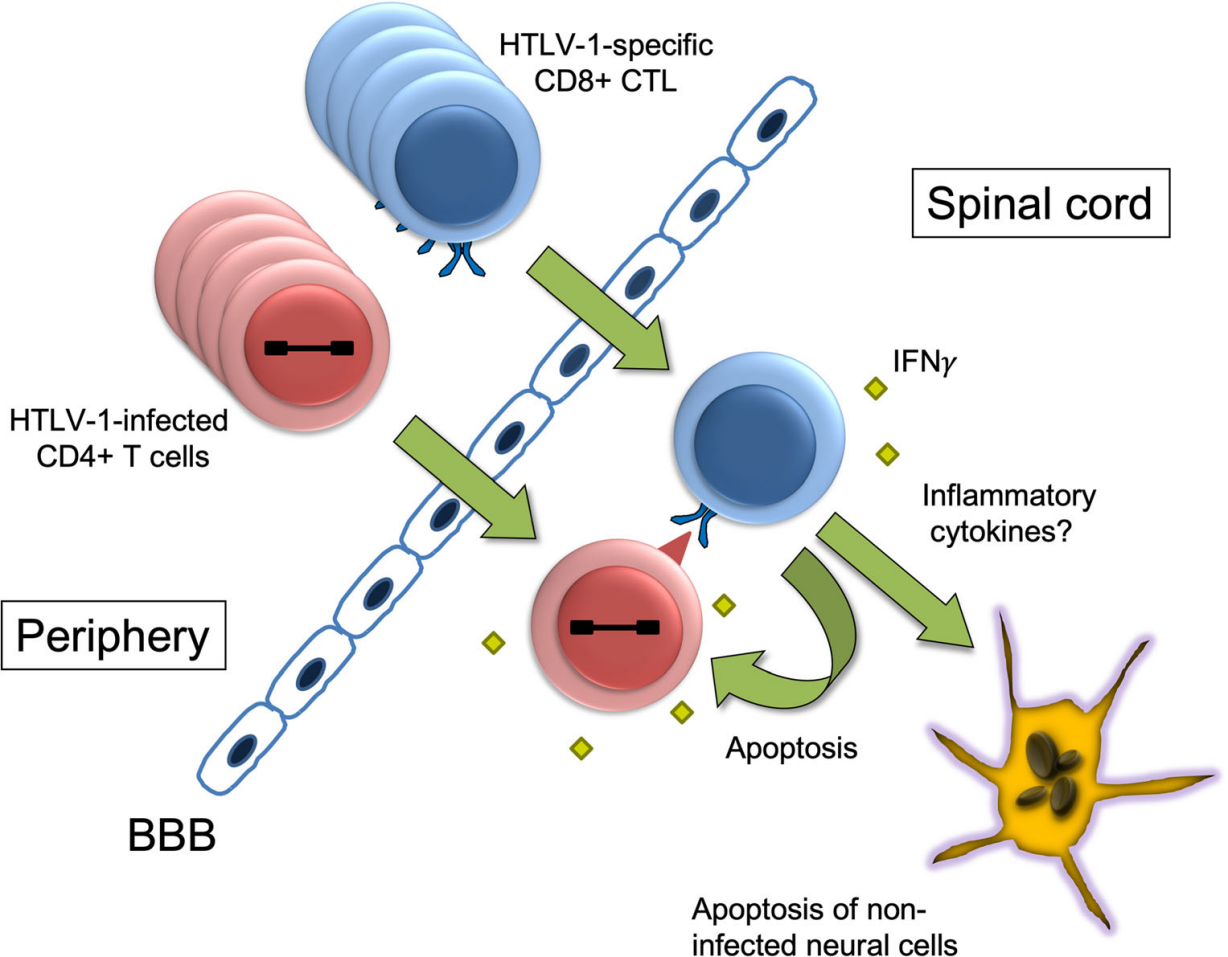
Proposed model of the immunopathology in human T-lymphotropic virus type 1 (HTLV-1)-associated myelopathy/tropical spastic paraparesis: bystander neural damage. HTLV-1-infected CD4^+^ T cells proliferate in the periphery and circulate in HTLV-1-infected individuals. The infected cells invade the central nervous system across the blood-brain barrier and express viral antigens. HTLV-1-specific cytotoxic T lymphocytes (CTL) are activated and expanded on stimulation by the HTLV-1 antigens and accumulate in the spinal cord. The CTL recognize viral antigens presented by human leukocyte antigen class I molecules on the infiltrating HTLV-1-infected CD4^+^ T cells, leading to the secretion of proinflammatory cytokines, such as interferon-γ and tumor necrosis factor-α. HTLV-1-specific inflammation mediated by the interaction of HTLV-1-infected CD4^+^ T cells with HTLV-1-specific CTL causes apoptosis in adjacent neural cells (bystander damage) in the CNS, resulting in tissue destruction and degeneration. BBB, blood-brain barrier

HTLV-1-specific CTL infiltrate the CNS and play a role in HAM/TSP pathogenesis (Kubota 2017). Activated HTLV-1-specific CTL is markedly increased in the periphery of patients with HAM/TSP. Additionally, abundant CD8^+^ T cells infiltrate the spinal cord and express the TIA-1 molecule, which is a CTL marker (Umehara et al. 1994). A recent study visualized HTLV-1 Tax-specific CTL infiltrating the CNS using an MHC/Tax tetramer (Matsuura et al. 2015). The frequency of HTLV-1 Tax-specific CTL is more than 20% in CD8^+^ T cells infiltrating the CNS. In addition, HTLV-1 proteins are not detected in the CNS-resident cells, but they were identified in more than 60% of the infiltrating CD4^+^ T cells. Although neurons were generally preserved, approximately 40% of CD4^+^ T cells and some oligodendrocytes underwent apoptosis. Apoptotic oligodendrocytes were frequently in contact with CD8^+^ T cells, resulting in demyelination. These findings indicate that the immune responses between HTLV-1-specific CTL and HTLV-1-infected CD4^+^ T cells could cause apoptosis in surrounding neural cells (bystander damage; Fig. 1). It will be necessary to elucidate the underlying mechanisms for how and why uninfected neural cells become apoptotic.

### Animal model

Animal models have been widely used to study HTLV-1 viral transmission, disease pathogenesis, and treatment (Niewiesk 2016). However, there is no accurate animal model of HAM/TSP, which has hindered the understanding of precise pathogenic mechanisms and development of effective treatments in the disease. Studies with rabbit, rat, and simian models have successfully led to the understanding of routes of transmission of the HTLV-1 virus, anti-viral immune responses, and the means of preventing its transmission (Lairmore et al. 2005). Wistar-King-Aptekman-Hokudai (WKAH) rats emerged as a model of HAM/TSP. HTLV-1-infected WKAH rats develop spastic paraparesis with degenerative thoracic spinal cord and peripheral nerve lesions after inoculation (Ishiguro et al. 1992; Kushida et al. 1993). However, lesions in humans showed a marked T cell infiltration of affected regions, while lesions in the rats did not. In addition, the HTLV-1 provirus has been identified in microglia/macrophages associated with lesions in rats, in contrast with humans (Kasai et al. 1999). Therefore, the pathogenesis of the rat model is considered to be different from that of human HAM/TSP. Mouse models have traditionally been cost-effective and easy to develop and maintain. Hence, mice have been manipulated to establish HTLV-1 infection through the generation of transgenic and humanized mice models. One such model is the Tax-transgenic mouse, which restricts Tax expression to developing thymocytes, demonstrating characteristic ATLL-like phenotypes (Hasegawa et al. 2006). Another common feature of Tax-Tg mice is the development of chronic arthritis at 2–3 months (Panfil et al. 2013) and a small portion (8/297) of Tax-Tg mice developed HAM/TSP-like disease with symmetrical paraparesis of the hind limbs though it was caused by the invasion of histiocytic sarcoma cells into the lumbar spinal cord (Ohsugi et al. 2013). Another model, the HBZ-transgenic mouse, expressed HBZ under the control of a CD4 promoter leads to inflammation in skin and lung as well as T cell lymphoma (Satou et al. 2011). However, currently, there is no report that HBZ-Tg mice develop inflammatory neurologic diseases and it remains unknown how tissue specificity of HTLV-1 associated inflammatory diseases are determined. A recent development is the use of humanized mice which, upon transfer of CD34^+^ human umbilical cord stem cells, generate human lymphocytes (Niewiesk 2016). Inoculation of immunodeficient mice with ATLL cells or HTLV-1 transformed cell lines provide an opportunity to investigate disease development and evaluate treatment for ATLL. Recently, humanized mice model using Balb/c-Rag1^−/−^γc^−/−^ and bone marrow-liver-thymic mouse showed establishment of peripheral infection that led to lymphocytic infiltration with concomitant Tax expression and resulting myelin disruption within the CNS of infected mice (Ginwala et al. 2017). In addition, upregulation in the expression of several immune checkpoint mediators such as PD-1, TIGIT, and Tim-3 was observed on CD8^+^ T cells in various organs including the CNS of infected hu-mice. This humanized mouse model could be a suitable model to evaluate the imunopathogenesis and develop a novel treatment for HAM/TSP.

### Treatment

In HAM/TAP, most therapeutic trials have aimed to inhibit or regulate the immune response, or to reduce the HTLV-1 proviral load in an attempt to decrease the risk or alter the course of the disease. Corticosteroids are most widely used to decrease the inflammation in the CNS, particularly in the early stage. Motor disability in some patients could be improved with steroids (Nakagawa et al. 1996; Croda et al. 2008), but improvement is typically not maintained and the drug is discontinued due to various side effects. IFN-α is a medication in which efficacy was demonstrated in randomized placebo-controlled trials (Izumo et al. 1996; Arimura et al. 2007); however, the therapeutic benefit is insufficient. Reverse transcriptase inhibitors, which are used to treat HIV-1 infection, were not effective against HTLV-1 in clinical trials (Taylor et al. 2006; Macchi et al. 2011). Recently, a humanized anti-CCR4 monoclonal antibody, mogamulizumab, effectively reduced both the PVL and inflammatory activity in cells obtained from patients with HAM/TSP (Yamauchi et al. 2015). Phase 1/2a clinical trials revealed the safety and short-term effectiveness of mogamulizumab in patients with HAM/TSP (Sato et al. 2018). Hu-Mikβ1, which is a humanized monoclonal antibody against the β subunit shared by the IL-2 and IL-15 receptors (IL-2/IL-15Rβ; CD122), has been reported to inhibit abnormal T cell proliferation and HTLV-1-specific cellular immune responses by blocking IL-15 action in HAM/TSP patients (Azimi et al. 1999; Enose-Akahata et al. 2008). The treatment with Hu-Mikβ1 showed the inhibition of aberrant CD8^+^ T cell function in HAM/TSP patients although no clinical efficacy was observed (Enose-Akahata et al. 2019). Raltegravir, which is an integrase inhibitor used for the treatment of HIV-1, was reported to reduce cell-free and cell-to-cell transmission of HTLV-1 in vitro (Seegulam and Ratner 2011). Since high PVL is known to be the main risk factor for developing HAM/TSP in infected subjects, PVL reduction is a reasonable therapeutic goal. The trial of raltegravir is currently underway.

## Conclusion

HTLV-1 preferentially infects CD4^+^ T cells and establish infected T cell clones in the host, regulating the balance between proviral latency and reactivation. Clonal expanded T cells infected with HTLV-1 dysregulate T cell function and differentiation and subsequently induce HTLV-1-specific T cell responses. These cell-mediated immune responses and released proinflammatory cytokines could cause neural damage, which is thought to play a central role in the development of HAM/TSP. Recent advances in research provide a better understanding of mechanisms associated with HTLV-1-infected cells and cellular immune responses in HAM/TSP. Further research will be required to develop strategies to eliminate HTLV-1-infected cells or enhance cellular immunity for the development of effective treatments in patients with HAM/TSP.
